# Risk of Kaposi's sarcoma and of other cancers in Italian renal transplant patients

**DOI:** 10.1038/sj.bjc.6602346

**Published:** 2005-01-25

**Authors:** D Serraino, P Piselli, C Angeletti, E Minetti, A Pozzetto, G Civati, S Bellelli, F Farchi, F Citterio, G Rezza, S Franceschi, G Busnach

**Affiliations:** 1 Dip. Epidemiologia, INMI L Spallanzani, Via Portunese 292, 00149, Rome, Italy; 2 Unità Nefrologia, Dialisi & Terapia Trapianto Renale, Osp Niguarda Ca' Granda, Piazza Ospedale Maggiore 3, 20162, Milan, Italy; 3 Clinica Chirurgica, Univ Cattolica Sacro Cuore, Policinico ‘A. Gemelli’, Largo A. Gemelli 8, 00168 Rome, Italy; 4 Dip Malattie Infettive, Istituto Superiore di Sanità, Viale Regina Elena 292, 00161 Rome, Italy; 5 International Agency for Research on Cancer, 150 cours Albert Thomas, 69372 Lyon cedex 08, France

**Keywords:** renal transplant, cancer risk, Kaposi's sarcoma, liver cancer, Italy

## Abstract

A follow-up study of 1844 renal transplant patients in Italy showed a 113-fold increased risk for Kaposi's sarcoma. Kaposi's sarcoma risk was higher in persons born in southern than in northern Italy. Significant increases were also observed for cancers of the lip, liver, kidney and for non-Hodgkin's lymphoma.

Renal transplant patients experience higher rates of cancer, particularly virus-related cancers, than the general population. The overall cancer risk is three- to four-fold elevated, but some specific cancers show greater increases (Kinlen, 1992). For nonmelanoma skin cancers and lip cancer, the relative risks are increased 10- to 60-fold (Kinlen *et al*, 1979; Birkeland *et al*, 1995; Adami *et al*, 2003), while the excess of non-Hodgkin's lymphoma (NHL) is about five- to 10-fold (Hoshida *et al*, 1997; Birkeland *et al*, 2000; Adami *et al*, 2003). Kaposi's sarcoma (KS) has been frequently reported in transplant patients in the United States (Penn, 2000), in the Middle East (Qunibi *et al*, 1988) and in Italy (Montagnino *et al*, 1996; Pedotti *et al*, 2003), but the excess risk has not been closely quantified. No increased risk of KS has been reported in transplant patients from the Nordic countries (Birkeland *et al*, 1995, 2000; Adami *et al*, 2003), or Japan (Hoshida *et al*, 1997).

The prevalence of infection with KS-associated herpesvirus (i.e., human Herpesvirus type 8 (HHV-8)) (Whitby *et al*, 1998; Serraino *et al*, 2000; Vitale *et al*, 2001) and the incidence of classic KS (Franceschi and Geddes, 1995; Dal Maso *et al*, 2004) are relatively high in Italy, particularly in the South. The aim of this study was to quantify the excess of KS risk, to identify factors associated with KS occurrence, and also to define the spectrum of cancers associated with immunosuppression in patients from two major Italian transplant centres.

## MATERIALS AND METHODS

Anonymous information was collected on 1844 patients resident in different parts of Italy and transplanted in northern (Milan) or central Italy (Rome). Of these, 1340 received their transplants at Niguarda Hospital, Milan, between 1972 and 2003 and 504 at Policlinico Gemelli Hospital, Rome, between 1970 and 2000. As with other investigations, we excluded patients who survived less than 10 days after transplant, those with a pretransplant history of cancer, or who developed cancer in the 30-day period after transplantation (Adami *et al*, 2003; Pedotti *et al*, 2003). Nonmelanoma skin cancers were not evaluated since information on basal cell carcinoma was not recorded and that on squamous cell carcinoma might not have been complete.

Person-years (PY) at cancer risk were measured from the date of first renal transplant and ended upon at tumour diagnosis, death, or date of last follow-up visit, whichever occurred first. For the purposes of this analysis, periods of graft failure were not considered. For each type of cancer observed, the expected number (standardised for sex, age, and area of residence) was computed using incidence rates from all population-based cancer registries of Italy for the 1988–1992 period (Parkin *et al*, 1997). Standardised incidence ratios (SIRs) were computed for all cancers with at least two observed cases, and were obtained by dividing the number of observed cases by the number of expected cases. Ninety-five percent confidence intervals (CI) of SIRs were determined using the Poisson distribution (Breslow and Day, 1980). Incidence rate ratios (IRRs) and their 95% CIs, adjusted for sex, age, area of birth, and time since transplant, were computed to assess the association between selected characteristics and KS risk among transplant patients.

## RESULTS

The median age of the 1844 renal transplant patients was 42 years (interquartile range: 31–51 years). In 15 940 PY of observation (median follow-up time: 7.0 years), 104 cases of cancer, other than nonmelanoma skin cancers, were diagnosed. Standardised incidence ratios according to cancer type or site, and sex are shown in Table 1.

The SIR for all cancers was 1.8 (95% CI: 1.5–2.2), and was of similar magnitude in the two sexes. The SIR for KS was 112.6 but it was greater among women (SIR=451, 95% CI: 147–1053) than men (SIR=93, 95% CI: 55–147). A 6.6-fold increased SIR (95% CI: 3.7–11.0) was seen for NHL. Other cancers with significantly elevated SIRs included: carcinomas of the lip (SIR=14.6; 95% CI: 1.8–52.7), liver (SIR=3.9; 95% CI: 1.5–8.6), and kidney (SIR=3.9; 95% CI: 1.6–8.0). The SIRs for lip and liver cancers were more elevated in women than men, whereas the excess risk for native renal cancer was found in men only (Table 1). Standardised incidence ratios for stomach (SIR=1.2), colon (SIR=1.0), lung (SIR=0.8), and female breast cancers (SIR=0.7) were close to unity (Table 1).

Among renal transplant patients, KS was more frequent (IRR=3.0, 95% CI: 1.1–8.5) in persons aged 50 years or older at transplant than in those younger than 40. Kaposi's sarcoma was nonsignificantly more common in men than in women, whereas it was nearly six-fold more frequent (95% CI: 1.6–19.7) in patients born in southern Italy, as compared to those born in northern Italy (Table 2). A strong inverse association emerged between KS occurrence and time since renal transplant. In the first year following transplant, KS was 4.5-fold more frequent (95% CI: 1.6–12.2) than after 4 or more years (Table 2).

Most of these 1844 patients (87%) received their transplant after cyclosporin came into widespread use (from 1983 onwards), and one-third after the introduction of the newer immunosuppressive drugs (from 1997 onwards). All 23 KS cases were diagnosed in 1983 or later inpatients who were treated with cyclosporin-containing regimens. No significant difference in KS IRRs was seen according to the calendar period of transplant (Table 2).

## DISCUSSION

Our study provides estimates of cancer incidence for renal transplant recipients in northern or central Italy and IRRs for KS. The overall increase in cancer risk we found (1.8-fold) is somewhat lower than that reported from previous investigations in transplant patients (Kinlen *et al*, 1979; Birkeland *et al*, 1995; Hoshida *et al*, 1997; Adami *et al*, 2003), but the exclusion in our analysis of nonmelanoma skin cancers explains a large part of this difference.

With respect to the increased risks for NHL and renal cancer, our results agree with previous investigations (Kinlen, 1992; Birkeland *et al*, 1995; Adami *et al*, 2003). It is noteworthy that all cases of renal cancer in this study involved the native kidney, a distinction not mentioned in previous studies. The quantified high risk of KS, particularly in women, and the peak in KS risk in the first year following transplant are new findings. The short induction period between transplant and KS diagnosis suggests that KS development in transplant patients is associated with a rapid re-activation of latent HHV-8 infection (Andreoni *et al*, 2001; Cannon *et al*, 2003). The investigation of geographical factors confirmed a higher (about six-fold) KS risk for individuals born in the South of Italy, as compared to those born in the North (Geddes *et al*, 1995; Dal Maso *et al*, 2004). On account of massive South-to-North migration in Italy in the last decades, 44.3% of our study patients born in the South lived in the North at time of transplant. The KS incidence rate among renal transplant recipients born and resident in the South (i.e., 24.16 per 10 000 PY) was about five-fold higher than that among those born and resident in the North (i.e., 4.62 per 10 000 PY) (data not shown in tables). Moreover, KS incidence among patients who were born in the South, but lived in the North, was similar to that of patients who were born and resident in the South (i.e., 24.70 per 10 000 PY). Information on age at migration was not available in this study, but the stronger association of KS with place of birth than with area of living suggests that acquisition of HHV-8 occurs early in life.

This study also revealed that renal transplant patients in Italy have a four-fold increased risk of liver cancer, an excess risk not generally seen in transplant patients elsewhere (Kinlen, 1992; Birkeland *et al*, 1995; Adami *et al*, 2003). In Italy, mortality rates for liver cancer (La Vecchia *et al*, 2000) and the prevalence of infection with hepatitis B virus and/or with hepatitis C virus among dialysis patients are high (Petrosillo *et al*, 1993; Fissell *et al*, 2004). The higher SIR for liver cancer found in our study than elsewhere may also be related to the fact that infection with hepatitis C virus in the recipient is not generally considered a contraindication for renal transplant in Italy.

In conclusion, our findings in patients who underwent renal transplantation in northern or central Italy provide further evidence of the strong association between organ transplant and virus-related cancers. Our observations on post-transplant KS confirm the need for HHV-8 screening protocols and for guidelines on the clinical management of HHV-8 infection in transplant centres, at least in areas endemic for KS (Serraino *et al*, 2005).

## Figures and Tables

**Table 1:** Standardised incidence ratio (SIR) of cancer and corresponding 95% confidence interval (CI) after renal transplantation, according to cancer site or type and sex (Italy, 1970–2003)

	**Total**			**Men**		**Women**	
**Cancer site/type (ICD-9)**	**Obs**	**SIR**	**95% CI**	**SIR**	**95% CI**	**SIR**	**95% CI**
Lip (140)	2	14.6	1.8–52.7	7.8	0.2–43.3	118.0	3.0–658
Stomach (151)	4	1.2	0.3–3.0	1.1	0.2–3.2	1.5	0.0–8.2
Colon (153)	4	1.0	0.3–2.5	0.7	0.1–2.6	1.6	0.2–5.9
Liver (155)	6	3.9	1.5–8.6	2.3	0.5–6.6	15.6	3.2–45.6
Pancreas (157)	2	1.6	0.2–5.6	2.0	0.3–7.3	NC	—
Larynx (161)	2	1.0	0.1–3.7	1.1	0.1–3.8	NC	—
Lung (162)	8	0.8	0.4–1.6	0.8	0.3–1.6	1.2	0.0–6.9
Melanoma (172)	2	1.4	0.2–5.0	1.1	0.0–6.1	1.9	0.1–10.6
Kaposi's sarcoma (KAP)	23	112.6	71.4–169	93.2	55.3–147	451.2	147–1053
Bladder (188)	3	0.7	0.1–2.0	0.5	0.1–1.8	2.9	0.1–15.9
Kidney (189)	7	3.9	1.6–8.0	4.8	1.9–9.9	NC	—
Thyroid (194)	2	2.4	0.3–8.5	5.8	0.7–20.8	NC	—
Non-Hodgkin's lymphoma (200+202)	15	6.6	3.7–11.0	6.1	3.0–11.2	8.1	2.6–18.8
Multiple myeloma (203)	2	3.8	0.5–13.7	2.6	0.1–14.5	6.9	0.2–38.2
Breast, female (174)	5	—	—	—	—	0.7	0.2–1.7
Cervix uteri (180)	2	—	—	—	—	2.8	0.3–10.1
Corpus uteri (182)	5	—	—	—	—	4.8	1.6–11.2
Ovary (183)	2	—	—	—	—	2.5	0.3–9.2
All cancers but skin^a^	99	1.8	1.5–2.2	1.8	1.4–2.3	1.8	1.2–2.6

Obs=observed number of cancers. NC=The SIR was not computed because no cancer cases were observed.

a It includes 99 persons with one or more cancer diagnoses (one person had three primary cancers and three persons had two primary cancers). It also includes one case each for: brain, mesothelium, nose or middle ear, prostate, small intestine, testis (seminoma), tonsil and myeloid leukaemia, for a total of 104 cancer diagnoses.

**Table 2:** Incidence rate ratio (IRR) for Kaposi's sarcoma and corresponding 95% confidence interval (CI) after renal transplantation according to selected characteristics (Italy, 1970–2003)

	**Study population**		**Kaposi's sarcoma**		
**Characteristic**	**No.**	**(%)**	**No.**	**IRR^b^**	**95% CI**
*Sex*					
Women	640	(34.7)	5	1	—
Men	1204	(65.3)	18	2.4	0.8–7.0
					
*Age at transplantation*					
⩽39	854	(46.3)	9	1	—
40–49	518	(28.1)	7	1.9	0.7–5.2
⩾50	472	(25.6)	7	3.0	1.1–8.5
					
*Area of birth* a					
North	697	(39.3)	3	1	—
Centre	382	(21.5)	4	2.7	0.6–12.3
South	696	(39.2)	15	5.7	1.6–19.7
					
*Area of residence* a					
North	1078	(58.6)	10	1	—
Centre	400	(21.7)	5	1.5	0.3–7.0
South	362	(19.7)	8	1.2	0.4–3.6
					
*Time since transplantation (years)*					
⩾04	1310	(71.1)	8	1	—
1–3	371	(20.1)	7	1.6	0.6–4.7
<1	163	(8.8)	8	4.5	1.6–12.2
					
*Calendar period at transplantation*					
1970–1982	234	(12.7)	0	0	—
1983–1996	988	(53.6)	20	1	—
1997–2003	622	(33.7)	3	0.4	0.1–1.5
					
*Centre*					
Milan	1340	(72.7)	13	1	—
Rome	504	(27.3)	10	1.7	0.7–4.2
					
*Donor status*					
Cadaver	1625	(88.1)	20	1	—
Living	219	(11.9)	3	0.7	0.2–3.2

a The sum does not add up to the total because of missing values.

b IRR adjusted for sex, age at transplantation, area of birth, and time since transplantation.

## Appendix A

### Other members of the Immunosuppression and Cancer Study Group

A Navarra, M Scuderi, P Vanacore (INMI ‘L Spallanzani’ IRCCS, Rome, Italy); B Longo, M Dorrucci (Istituto Superiore di Sanità, Rome, Italy); MP Carrieri (INSERM U379, Marseille, France); C Pradier (Departement de Santé Publique, Hopital de l'Archet, Nice, France); ML Broggi, V Colombo, ML Perrino, L Radaelli (Ospedale Niguarda Cà Granda, Milan, Italy); L Dal Maso, J Polesel (Centro di Riferimento Oncologico, IRCCS, Aviano, Italy); M Castagneto (Università Cattolica, Rome, Italy).
